# The Application of Mucoadhesive Chitosan Nanoparticles in Nasal Drug Delivery

**DOI:** 10.3390/md18120605

**Published:** 2020-11-29

**Authors:** Soojin Shim, Han Sang Yoo

**Affiliations:** 1 Department of Infectious Diseases, College of Veterinary Medicine, Seoul National University, Seoul 08826, Korea; shimsj@iis.u-tokyo.ac.jp; 2 BioMax/N-Bio Institute, Seoul National University, Seoul 08826, Korea

**Keywords:** chitosan nanoparticles, nasal vaccines, mucosal delivery system

## Abstract

Mucosal delivery of antigens can induce both humoral and cellular immune responses. Particularly, the nasal cavity is a strongly inductive site for mucosal immunity among several administration routes, as it is generally the first point of contact for inhaled antigens. However, the delivery of antigens to the nasal cavity has some disadvantages such as rapid clearance and disposition of inhaled materials. For these reasons, remarkable efforts have been made to develop antigen delivery systems which suit the nasal route. The use of nanoparticles as delivery vehicles enables protection of the antigen from degradation and sustains the release of the loaded antigen, eventually resulting in improved vaccine and/or drug efficacy. Chitosan, which exhibits low toxicity, biodegradability, good cost performance, and strong mucoadhesive properties, is a useful material for nanoparticles. The present review provides an overview of the mucosal immune response induced by nanoparticles, recent advances in the use of nanoparticles, and nasal delivery systems with chitosan nanoparticles.

## 1. Introduction

Mucosal membranes line internal tissues of the body including respiratory, gastric, and genital tracts. The mucosal system has a broad recognition system which is called the “common mucosal immune system”; the stimulation of a local mucosal immune response could induce systemic mucosal immune response [1]. Therefore, using this common mucosal immune system, inducing an antigen-specific immune response throughout the body could be strategically advantageous for developing mucosal vaccines. In the upper respiratory tract, the nasal-associated lymphoid tissue (NALT) has an important role in induction of the mucosal immune responses [2]. It contains microfold cells (M cells) that phagocyte and transport antigens across the mucosal membrane [3]. NALT also contains immunocompetent cells such as T cells and B cells that are mainly involved in antigen recognition and antigen presentation [4]. Nasal vaccination offers various advantages compared to the parenteral strategy, such as the need for only a small dose of antigen, increased patient compliance, and stimulation of systemic mucosal immune responses.

However, the induction of the mucosal immune response remains a challenge because antigens need to overcome rapid clearance in the mucosal membrane and reach the inductive site of the mucosal immune system. In the field of drug delivery, the research on developing nanoparticles using biopolymers to overcome the mucosal barrier and optimize the effective concentration of antigens in the body is highly prolific [5]. Chitosan is a polysaccharide polymer produced by alkaline deacetylation of chitin [6]. It has a positive charge that may interact with negatively charged mucosal surfaces [7]. In addition to the mucoadhesive property, the use of chitosan nanoparticles (CNs) in nasal delivery could be a good option as they have some advantages for a mucosal delivery system including biocompatibility, sustained release of loaded drug and/or antigen, a wide range of applicable conditions, high stability, and low toxicity [8]. This review provides an overview of the induction of mucosal immune response by nanoparticles, recent advances in nanoparticles, and a mucosal delivery system via the nasal cavity using CNs.

## 2. Mucosal Delivery System

Most infections by pathogens occur at or through mucosal surfaces. For an ideal vaccine, there are some expected attributes that can prevent the pathogen from initial attachment, colonization of the mucosal epithelium, and replication in the mucosa [5]. Mucosal immunization with appropriate antigens can induce both humoral and cellular immune responses throughout the body [1,9]. To develop a mucosal vaccine targeting particular systems, an antigen delivery system must be be considered.

### 2.1. Induction of Mucosal Immunity

Organized inductive sites of mucosal immunity are in areas where pathogens and commensal bacteria are most likely to enter the body [1]. The aggregations of mucosal lymphoid follicles are called mucosa-associated lymphoid tissue (MALT), which comprises the mucosal immune system that can function independently of the systemic immune system [10]. In addition to NALT, MALT is composed of bronchial-related lymphatic tissue (BALT) and intestinal-related lymphatic tissue (GALT) [1]. The follicle-associated epithelium contains M cells that induce transcytosis of antigens across the epithelium to underlying mucosal cells such as B cells and dendritic cells (DCs) [11].

MALT contains DCs, macrophages, T cells, and B cells [11,12]. These are immunocompetent cells that are responsible for generating the antigen-specific immune response. An antigen is transported to the NALT or Peyer’s patches via M cells [5,13]. Antigen-presenting cells (APCs) process and present antigens to T cells in these lymphoid tissues. Chemokine–chemokine receptor interactions, such as those between chemokine ligand (CCL) 20 and chemokine receptor (CCR) 6, are involved in the antigen presentation of APCs to T cells [14]. Then, naïve T cells generate antigen-specific T cell subsets, including Th1, Th2, Th17, and cytotoxic T cells [10,15]. CD4+ T cells that are stimulated by DCs also induce IgA-committed B cell development including IgA class switching and affinity maturation in the germinal center [16]. Then, B cells migrate from the NALT and Peyer’s patches to the cervical and mesenteric lymph nodes, respectively [13]. Activation of cytokines, including transforming growth factor (TGF)-β, IL-2, IL-4, IL-5, IL-6, and IL-10, is involved in differentiation of IgA-producing B cells into plasma cells [13,17]. IgA-producing plasma cells subsequently produce dimeric or polymeric forms of IgA. Dimeric IgA becomes secretory IgA by binding to polymeric Ig receptors (pIgR) on the epithelial cells of the mucosal membranes and are released into the mucosal tract [3,18,19].

### 2.2. Mucosal Administration Route

The traditional mucosal administration routes are oral and nasal routes, and the immune induction sites differ according to immunization route [5,20]. Oral immunization is effective in inducing the immune response in the gastrointestinal tract, salivary glands, and mammary glands, while intranasal immunization is effective in the respiratory, gastric, and genital tracts [21,22]. These broad recognition systems are called the “common mucosal immune system” [18,19]. The production of IgA upregulates the expression of adhesion molecules for specific tissues and chemokine receptors that can induce homing of lymphocytes back to mucosa throughout the body [16,19]. For example, CCL28, the ligand for CCR10, is expressed in epithelial cells from several tissues, including the intestines, salivary glands, tonsils, upper and lower respiratory tract, and mammary glands [23]. Activated IgA-secreting B cells in mucosa express CCR10 so that CCR10 + IgA + B cells are attracted to these molecular expressing epithelial cells and induce systemic specific IgA responses throughout the body [19,24]. Secreted mucosal IgA intercepts, excretes and neutralizes antigens [25]. In addition, the production of serum IgA and IgG can also be induced by local immunization. The B cells activated in mucosa express peripheral homing receptors such as α4β1-integrin and leukocyte (L)-selectin, and these B cells can be spread throughout the body, producing antigen-specific immunoglobulin [19]. These mechanisms are advantageous for vaccination strategies for mucosal immunity as well as host defense since the appropriate activation of local mucosal immunity can induce systemic immune responses. As a well-known mucosal vaccine, an oral vaccine for poliovirus has been shown to induce mucosal IgA and systemic IgG production, and its efficacy is over 90% worldwide [21]. Other mucosal immunization routes are the rectal, vaginal, pulmonary, conjunctival, sublingual, and transcutaneous routes. The advantages, disadvantages, and the inductive sites of mucosal immunity according to these routes are summarized in Table 1.

Among the immunization routes, the nasal route has been preferred, since the nasal mucosa is a layer consisting of specialized antigen-sampling M cells overlaying the NALT [4]. The well-organized lymphoid tissue contains immunocompetent cells, including DCs, T cells, and B cells, that play key roles in the induction of an immune response in the URT [17]. Th0 cells are shown to be involved in NALT from naïve mice [2]. Therefore, after M cell uptake of the inhaled antigen, the immunocompetent population within NALT can induce T helper cell subsets corresponding to the antigen. In addition to the existence of NALT, the nasal route is considered an attractive route for administration, with the following advantages. Intranasal immunization requires fewer antigen doses than the parenteral and oral routes because the nasal cavity shows relatively less enzymatic degradation of antigens [30]. Another study showed that intranasal immunization induces less rapid but longer-lasting mucosal and serum antibody kinetics than oral immunization in humans [31]. The uptake of antigen into the blood circulatory system after intranasal immunization is shown to be relatively fast [32]. Intranasal immunization also led to an increase in antigen-specific lymphocyte proliferation, cytokine production, and induction of specific IgA antibodies [33,34,35]. Despite these advantages of nasal vaccines, there are several limitations, such as rapid mucociliary clearance and the enzymatic barrier, which interferes with the uptake of vaccines. Therefore, the delivery system of an antigen must be considered, since successful development of nasal vaccines depends largely on the vaccine delivery to the nasal mucosal surface [36].

### 2.3. Nanoparticles in Vaccine Delivery

**Figure 1:**
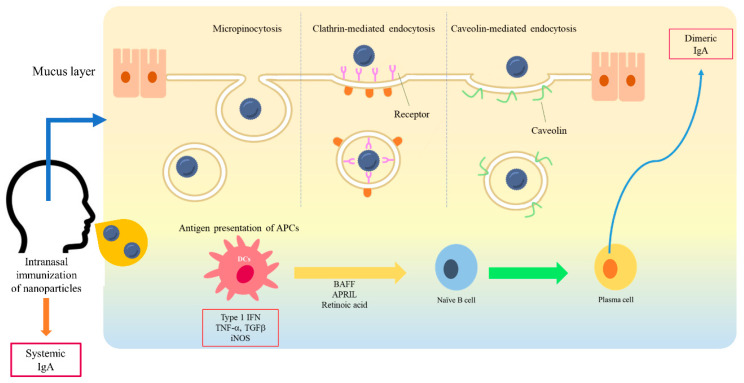
A schematic diagram of interaction between nanoparticles and a mucosal membrane, and the induction of mucosal immunity. At the mucosal membrane, the transport of nanoparticles is considered as mediated by caveolin-1, clathrin, and micropinocytosis into M cells. After antigen presentation, B cells migrate from the NALT to the cervical lymph nodes. IgA-producing plasma cells subsequently produce dimeric or polymeric forms of IgA. Dimeric IgA becomes secretory IgA on the epithelial cells of the mucosal membranes and is released into the mucosal tract.

Drug delivery is a method or process in which a pharmaceutical compound is administered for a higher therapeutic effect [26]. To improve the safety and efficacy of drugs or antigens, several drug delivery systems have been formulated using natural product-based carriers, including liposomes, micelles, and polymeric nanoparticles [27,37,38]. The internalization mechanisms of nanoparticles are different according to particle size, surface charge, loaded antigen, and types of cells [39,40,41]. A wide variety of polymers, including poly (D,L-lactide-coglycolide) (PLGA), poloxamers, chitosan, alginate, liposomes, and hydrogels, have been used for drug delivery in the mucosal surface [42]. The higher intestinal transport of smaller (~300 nm) particles than larger particles via enterocytes and M cells has been observed in vivo [43]. The size of <1 µM is reported as the preferred particle size for uptake by M cells [42]. The size of particles impacts cellular uptake and accumulation rates in the tissue due to its influence on the adhesion strength between nanoparticles and cellular receptors [44]. As for M cells, the transport of nanoparticles is considered to be mediated by many mechanisms of endocytosis. Several studies suggest that caveolin-1 [45] and clathrin [46] play a crucial role in the entry of nanoparticles into M cells. Another study suggests that nanoparticle endocytosis of M cells is most likely micropinocytosis [47]. For some nanoparticles, Toll-like receptor (TLR)-mediated stimulation has been also shown in several studies [39,48,49,50]. The previous literature in this field remains controversial but some suggest that the large size of nanoparticles may directly associate with TLRs [50]. On the other side, others suggest that the nanoparticle may act as a binding protein molecule due to its size and then the complex activates further TLR signaling pathways [51]. M cells have been shown to have the ability to discriminate between different commensal bacteria and modify subsequent immune responses [52]. Physicochemical properties of nanoparticles, including size, shape, surface potential, and hydrophobic/hydrophilic balance, may exert their effects on the mechanism selection of nanoparticle uptake of M cells (Figure 1).

The design of nanoparticle size is crucial to provide the best effects on cellular uptake and pharmacokinetics. Additionally, surface modification of nanoparticles alters ligand specificity and availability to interact with APCs [53]. Positively charged particles also exhibit enhanced mucoadhesive properties over negatively charged particles [54]. Conjugation of CD47 on the surface of nanoparticles minimized cellular uptake and enhanced their functionality to expand antigen-specific T cells [55]. Nanoparticles have the potential for many biomedical applications according to their specific properties. The nanoparticles used in mucosal delivery are summarized in Table 2.

## 3. Chitosan Nanoparticles in Drug Delivery

Chitosan, the partially deacetylated form of chitin, is one of the most widely used materials for biomedical delivery vehicles [54]. CNs have been widely used in the non-parenteral drug delivery of antibiotics, proteins, and peptide drugs, and vaccines for the treatment of cancer, pulmonary or gastrointestinal diseases, and brain and ocular infections [73]. Nanoparticles prepared with chitosan possess a mucoadhesive property with a positive charge and release the drug in a sustained manner [74,75]. Moreover, they can easily control the release rate of antigens by modification of the synthesis process. Previous studies have shown that CNs are effective delivery systems for mucosal vaccines, especially nasal vaccines, since they enhance mucosal absorption and have adjuvant activity in the mucosal membrane [26,54,76]. CNs are suitable for delivery vehicles since chitosan is well known to exhibit biocompatibility [77], non-toxicity [78], antimicrobial activity [79], gel and film forming abilities [80], immune-stimulatory functions [81], and hemocompatibility [82], as well as mucoadhesiveness [83].

### 3.1. Formulation of Chitosan Nanoparticles for Drug Delivery

CNs for vaccine delivery can be formulated by several different methods including ionic gelation, microemulsion, emulsification solvent diffusion, and polyelectrolytic interaction [84]. The most common method for preparing the antigen-loaded CNs is the ionic gelation method that induces spontaneous self-assembly of oppositely charged materials between cationic chitosan and anionic crosslinking substrates such as tripolyphosphate (TPP) or sodium sulfate [8]. This method is preferred because the chemical nature of the components remains unaltered and it shows less toxicity than chemical crosslinking [85]. The average diameter of the CNs is reported to be strongly dependent on the initial chitosan concentration, the degree of deacetylation (DDA) of the chitosan, and the presence or absence of salts in the medium [86]. The DDA of chitosan is usually between 70–95% and the molecular weight ranges from 10–1000 kDa [87]. To design suitable CNs for mucosal delivery, the properties of biodegradability, mucoadhesiveness, internalization rate, pH sensitiveness, release rate of loaded antigen, and adjuvant activity need to be considered.

Chitosan has been found to be biocompatible since it can be metabolized by human enzymes including lysozyme [77]. Furthermore, its amino and hydroxyl groups make chemical modification easy. The interaction between chitosan and mucosal membranes is shown to be electrostatic and it can be adjusted by modifying the DDA and molecular weight of chitosan [88]. The higher molecular weight of chitosan induces stronger adhesion [89]. The charge density of chitosan-based particles depends on the DDA and also affects the mucoadhesiveness [83]. In intestines, adhesion of chitosan microspheres was stronger when the density of cross-linking of chitosan was low since the number of free amino groups in chitosan was increased [90]. In addition, the interaction between chitosan and mucosal membrane was increased at acidic pH levels [90].

The particle size of CNs could be adjusted by concentration or molecular weight of chitosan. Increasing chitosan concentration or molecular weight generates intermolecular hydrogen bond (−OH) and intermolecular electrostatic repulsion with −NH3+ on the chitosan surface in a balanced manner and increases the size of CNs, whereas it decreases with the increase of DDA [75,83,91]. Generally, nanoparticles between 30–60 nm can bind to cellular receptors and drive the membrane-wrapping process without a receptor shortage affecting endocytosis [92]. On the other hand, larger nanoparticles can be loaded with larger antigens and accumulate in the liver and spleen more rapidly [93].

DDA also influences the immunotoxicity of produced CNs. In a study which compared immunotoxicity with different DDA of CNs, lower DDA CNs (80%) were more cytotoxic for human peripheral blood monocytes and increased reactive oxygen species (ROS) production in the murine macrophage cell line, RAW 264.7 cells, compared to higher DDA CNs (93%) [94].

Despite many applicable possibilities, chitosan exhibits a pH sensitive property; it easily dissolves at lower pH while it is insoluble at higher pH. The pH sensitivity of chitosan could limit its application in antigen delivery as many proteins are not stable at low pH. Therefore, modification of chitosan, including making the particles, coating with antigens, and adding derivatives, is necessary to apply chitosan as a delivery agent.

### 3.2. Adjuvant Activity of Chitosan Nanoparticles

Early studies showed that the CNs formed by mixing two polyelectrolytes carrying complementary charges at alkaline pH are biologically compatible with the mucosal surface, thereby being applicable carriers for nasal administration [37,95]. Positively charged CNs not only have mucoadhesive properties but also promote the internalization rate and cellular uptake compared to negatively and neutrally charged CNs [96]. CNs are shown to decrease the clearance of components from the nasal cavity, which eventually may lead to crossing the epithelial barrier and uptake by M cells [97]. CNs have also been reported to have immune-enhancing effects as adjuvants by activating macrophages and polymorphonuclear cells and inducing cytokines [6,7,98]. Nevagi and coworkers showed that the stimulation of protein-conjugated CNs induced DC differentiation and macrophage activation in an in vitro study [99]. Coating chitosan led to a higher interaction with Caco-2 cells compared to coating with polyethylene glycol (PEG), showing that chitosan exhibits good compliance with mucosal epithelial cells, whereas it showed a limited uptake in THP1 cells [100]. In the comparison of ion absorption by chitin nanofibers (CNFs) and CNs, absorption efficiency for CNs was greater than CNF [101]. In a comparison of drug delivery in breast cancer cell line, MCF-7 cells, between chitosan-polymerized graphene oxide and polyvinylpyrrolidone-polymerized graphene oxide nanoparticles, a chitosan nanocarrier was more suitable for application since it increased drug loading capacity and greater inhibition of MCF-7 cell lines [102]. Transcriptomic analysis revealed that intranasal immunization of CNs to mice induced cellular movement of lymphocytes, complement activation, fever, and production of cytokines within NALT [103]. Furthermore, intranasal immunization with chitosan alone could fully protect BALB/c mice from a highly pathogenic H7N9 virus by stimulating the innate immune system. The significant infiltration of leukocytes and the levels of proinflammatory cytokines were observed in the lungs of immunized mice compared with those in untreated groups [104]. Mucosal immunity and protective efficacy of an intranasally delivered influenza vaccine was improved by CNs [81]. By using these immune-enhancing effects, CNs have been demonstrated as adjuvants for some antigens, including DNA [56,105], toxin [36], and ovalbumin [106], and could be vaccine candidates against *Escherichia coli* O157:H7 [107], *Bacillus anthracis* [57], *Chlamydia psittaci* [108], *Mycobacterium tuberculosis* [109,110], *Brucella abortus* [103,111,112], Hepatitis B virus [113,114], and influenza viruses [115,116].

### 3.3. Chitosan Nanoparticles for Nasal Vaccines

CNs have been used in mucosal delivery, especially in nasal delivery, as mucosal vaccine adjuvants in mice, rabbits, chickens, pigs, and cattle [66,105,110,117,118,119]. In several studies, intranasal immunization with CNs induced both cellular and humoral immune responses. In a study conducted by Li et al., a *Chlamydia psittaci* vaccine loaded with CNs induced Th1 immune responses in mice. They also compared the induction of mucosal immunity according to the immunization route of intranasal, intramuscular, and simultaneous immunization of antigen-loaded CNs. As expected, simultaneous immunization mediated stronger humoral responses to the intranasal and intramuscular immunization strategies alone, but nasal IgA and vaginal IgA levels were comparable to the intranasal route, suggesting that intranasal immunization has a more pronounced increase in humoral and mucosal immunity than intramuscular immunization [108].

**Figure 2:**
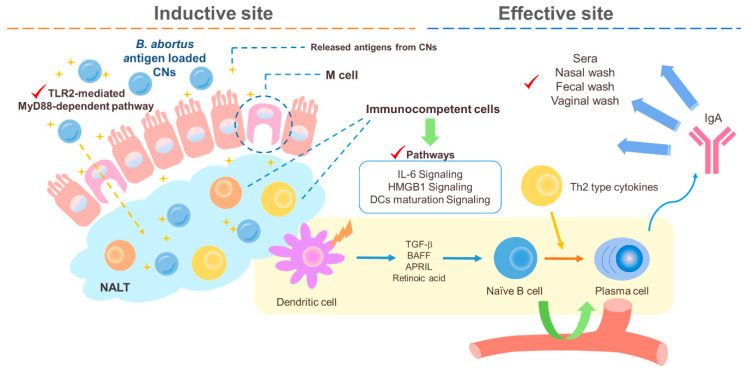
A schematic diagram of the suggested pathways of the immune response induced by *B. abortus* recombinant protein loaded CNs (chitosan nanoparticles). Loading *B. abortus* antigen, Mdh, into the CNs induced enhanced transport of Mdh in the in vitro M cell model and CNs–Mdh triggered activation of HMGB1, IL-6, and DCs maturation signaling within NALT in BALB/c mice and elicited Th2-related response with production of IgA at 6wpi (weeks post infection).

The intranasally delivered CNs and *Brucella abortus* antigen in mice were found to induce mixed Th1/Th2 responses at 4 weeks post infection (wpi) and then finally to induce a Th2 response at 6 wpi [112]. Our previous studies revealed that malate dehydrogenase (Mdh), a promising *B. abortus* antigen, induced enhanced transport of Mdh when loaded in CNs in the in vitro M cell model and that CN-Mdh triggers signaling pathways of HMGB1, IL-6, and DC maturation within NALT in BALB/c mice [103,111]. Using these delivery systems, three *B. abortus* antigen (Mdh, Omp10, and Omp19)-loaded CNs elicited each antigen-specific IgA with a Th2-polarized immune response [120] (Figure 2). The combination of these highly immunogenic antigens elicited IgG specific to each type of antigen and IgA specific to the Mdh. Considering that the loading efficiency (LE) and release rate of antigen-loaded CNs were different based on the antigen, making a cocktail based on the property of each antigen-loaded CN will enhance the multiplicity of the antibody for mucosal and systemic immune responses.

The *Streptococcus equi* extract with CNs or liposomes via nasal delivery induced both Th1 and Th2 responses [66]. Increased IgA in the lungs was significantly observed in the CN-loaded group, which is probably due to their different mucoadhesive properties. The delivery of CN-loaded antigens has been shown to elicit specific mucosal immune responses in serum and in mucosal fluid, including nasal, saliva, bronchoalveolar lavage, lung, and vaginal secretions. Intranasal immunization with CNs loaded with DNA expressing the *Streptococcus pneumoniae* surface antigen A (PsaA) elicited enhanced mucosal and systemic antibody production compared with immunization with DNA alone [121]. CNs loaded with hemagglutinin (HA)-split influenza virus protein were shown to induce higher mucosal and systemic antibody titers than antigen alone [59].

Moreover, CNs promote antigen internalization by APCs and enhance the nasal residence time of an antigen. For instance, in a study that compared two nanoparticles, CNs and CNs plus alginate, as adjuvants for mast cell activator compound 48/80, nanoparticles of higher amounts of chitosan were better internalized by macrophages and dendritic cells and enhanced the residence time of C48/80 at the nasal membrane. The intranasal immunization of mice with *Bacillus anthracis* protective antigen (PA) adsorbed on C48/80 CNs elicited significant serum anti-PA antibodies and better balanced the Th1/Th2 profile compared to CNs plus alginate-C48/80 or C48/80 alone [118]. This observation correlated closely with the amount of chitosan, and the authors suggest that a reduced amount of chitosan could reduce mucoadhesive property, which may have hindered the uptake of the antigen. The recent studies in which CNs were used in nasal delivery are summarized in Table 3.

### 3.4. Application of Chitosan in Industry and Limitation of Using Cns Nasal Vaccines

Currently, the polysaccharide is classified by U.S. FDA as Generally Recognized As Safe (GRAS) for food [123]. As for its derivative, to date, chitosan has been applied in food [124], cosmetics [125], textiles [126], contact lenses [127], wound healing [128], reduction of dental plaque formation [129], implants [130], and tissue engineering [131]. Specifically, chitosan has been used to prevent or treat wound and burn infections because it is antimicrobial, nontoxic, biocompatible, and is able to deliver extrinsic antimicrobial agents to wounds and burns [132]. As wound dressers, various chitosan-based products are commercially available in the market, including ChitoGauze^®^ OTC, HemCon^®^ Bandage, ChitoFlex^®^ PRO, Chitodine^®^, and Celox™. As CNs have many advantages including biocompatibility, encapsulation of antigens, stabilization, controlled release of antigens, wide range of applicable conditions, intracellular persistence in APCs, induction of systemic mucosal immunity, and suitability for intranasal immunization, several efforts are being made to develop antigen delivery systems using CNs. Nevertheless, evidence of the effect of their properties on clinical use remains lacking. CNs have some drawbacks including low solubility in neutral and alkaline pH [54], different preparation protocols according to the loading antigen, and difficulty in changing the pore size of nanoparticles [133]. To overcome these problems, several attempts such as changing DDA and molecular weight, adding derivatives, improving adhesiveness, and increasing circulation time in blood are feasible as long as the loaded antigen is stable. Although low toxicity in animal models is shown in chitosan, its real impact on the delivery system remains extremely limited. Further mechanistic studies and cooperation between researchers in materials science, immunology, and bioengineering are needed to determine the physicochemical properties of the specific antigen to be encapsulated and to apply CNs to their intranasal immunization as vaccine adjuvants.

## Figures and Tables

**Table 1:** Mucosal effector sites associated with immunization routes.

Route	Advantages	Disadvantages	Inductive Site for Iga Antibody Responses	Ref.
**Nasal**	Primary invasive site for airborne pathogens Easy access to mucosal immune induction sites permits less antigen compared with oral administration	Degradation of antigen by host Adjuvants are required	Upper respiratory tract Lower respiratory tract Reproductive tract Blood	[2,26,27]
**Oral**	Induction of mucosal immune responses to other sites through the mucosal immune network Primary invasive site for airborne pathogens	Degradation of antigen by gut digestive process and bacterial proteases High doses required	Stomach Small intestine Colon Blood	[26,28]
**Vaginal**	May be advantageous for sexually transmitted diseases	Poor patient compliance Poor induction of both systemic and vaginal mucosal immune responses Strong adjuvants are required	Reproductive tract Blood	[1,29]
**Rectal**	May be advantageous for sexually transmitted diseases	Poor patient compliance for immunization Strong adjuvants are required	Colon Rectum Blood	[1,9]

**Table 2:** Nanoparticles used in drug delivery systems.

Polymeric Materials	Size (nm)	Route	Antigen	Descriptions	Ref
Chitosan	200	Intramuscular	*ptfA* gene of *Pasteurella multocida*	Elicited significant IgG response in chicken. Conferred partial protective immunity against *P. multocida* challenge.	[56]
Chitosan	254	Intra-muscularperitoneal	*Bacillus anthracis* toxin, protective antigen (PA)	Elicited significant IgG (IgG2a dominantly) response in mice. Conferred ~83% protective rate against *B. anthracis* challenge.	[57]
Chitosan	228 −399	Oculo-nasal	Inactivated bronchitis virus	Elicited significant specific IgA and IgG response in mice.	[58]
Chitosan	300 −350	Nasal	Influenza hemagglutinin	Elicited the significant IFN-γ-secreting cells in spleens of mice. Conferred 100% protective rate against influenza virus challenge.	[59]
Chitosan	116.6	Oral	*E. coli* O157:H7 recombinant EIT and STX toxin	Elicited significant specific IgA and IgG response in mice. Conferred partial protective immunity against *E. coli* O157:H7 challenge	[60]
PLGA	300	Subcutaneously	*Leishmania* antigens	Elicited significant IgG (IgG2a dominantly) response in mice. Conferred high protective rate against *L. infantum* challenge.	[61]
PLGA	633	Intraperitoneal	*Bacillus anthracis* toxin, PA	Elicited significant IgG response in mice. Conferred partial protective immunity against *B. anthracis* challenge.	[62]
Dendrimer	500	Intramuscular	mRNA replicons	Conferred partial protective immunity against influenza virus challenge in mice.	[63]
Liposomes	60 −120	Intranasal	Respiratory syncytial virus glycoproteins	Elicited higher levels of interferon-γ and long-term memory in mice.	[64]
Liposomes	<200	Oral	lipid-core peptide	Elicited specific IgA and IgG response in mice.	[65]
Liposomes	220	Intranasal	*Streptococcus Equi* antigens	Elicited significant IgA and IgG response in mice.	[66]
Liposomes	<200	Intranasal	Highly conserved B and T cell epitope peptides	Elicited significant IgA response in pig. Conferred partial protective immunity against swine influenza A virus challenge.	[67]
Liposomes	50 /100 /1000	Intranasal	Ovalbumin (OVA)	Elicited specific IgA and IgG (IgG1 dominantly) response in mice.	[68]
Silica	150	Intramuscular	Avian Leukosis Virus gp85 protein	Elicited significant IgG response in chicken. Conferred higher protective immunity against avian leukosis virus challenge.	[69]
Silica	<1000	Intratracheal	H1N1 influenza hemagglutinin antigen	Elicited specific IgA and IgG response in mice.	[70]
Inorganic nanoparticles (Gold)	12	Intranasal	Ion channel membrane matrix protein 2	Elicited specific IgG response in mice. Conferred 100% protective rate against influenza virus challenge.	[71]
Inorganic nanoparticles (Gold)	<50	Subcutaneously	EHEC-specific immunogenic antigens	Elicited specific IgA and IgG response in mice.	[72]

**Table 3:** Chitosan nanoparticles (CNs) for nasal vaccines.

CNs	Size (nm)	Antigen	LE (%)	Animal	Immunoglobulin		Ref
					IgA	IgG	
Chitosan nanoparticles	276.1	*Chlamydia psittaci* antigens	71.7	Mice	Elicited (specific); nasal wash and vaginal secretions	Elicited (specific); IgG1 dominantly	[108]
Chitosan nanoparticles	326.3/475.4/360.8/439.5	*Brucella abortus* antigens	51/78/ 71/72	Mice	Elicited (specific); nasal wash, fecal wash, vaginal secretions, and serum	Elicited (Specific); IgG1 dominantly	[120]
Chitosan nanoparticles	350–400	pHSP65pep gene of *Mycobacterium tuberculosis*	−	Mice	Elicited (specific); lung fluids	Elicited (Specific); IgG2a dominantly	[110]
Chitosan nanoparticles	500	Mast cell activator compound 48/80	18.65	Mice	Elicited (specific); nasal wash, fecal wash, vaginal secretions, and serum	Elicited (specific); IgG1 dominantly	[118]
Chitosan nanoparticles	571.7	Killed swine influenza antigen	67	Swine	Elicited (specific); nasal wash, bronchoalveolar lavage fluids, and lung lysates	Elicited (specific)	[81]
Chitosan nanoparticles	581.1	Influenza virus, CpG oligodeoxynucleotide, and *Quillaja* saponins	33.7	Rabbit	Elicited; nasal washes	Elicited	[115]
Human serum albumin conjugated Chitosan nanoparticles	290	pCMVluc and HBsAg gene of hepatitis B virus	−	Mice	Elicited (specific); nasal wash and vaginal secretions	Elicited (specific)	[113]
Chitosan/ N-trimethyl-aminoethyl-methacrylate chitosan (TMC) nanoparticles	141.3/139.4	Influenza A H1N1 antigen	88.5/Approximately 100	Mice	Elicited (specific); nasal wash, saliva, and lung wash	Elicited (specific)	[116]
TMC nanoparticles	365.2	*E. coli* O157:H7 recombinant EIT	−	Mice	Elicited (specific); fecal wash, eye wash, and serum	-	[60]
TMC liposome-based nanoparticles	280	B cell epitope derived from Group A streptococcus M-protein	97	Mice	Elicited (specific); saliva wash	Elicited (specific)	[122]
Chitosan-coated PLGA nanoparticles	500	Plasmid DNA of foot-and-mouth disease virus	−	Cattle	Elicited (specific); nasal wash and serum	Elicited (specific)	[105]
Chitosan-coated PLGA nanoparticles	819	Hepatitis B virus surface antigen (HBsAg)	62–67	Chicken	Elicited (specific); Serum	Elicited (specific)	[114]
Curdlan sulfate–*O*-(2-hydroxyl) propyl-3-trimethyl ammonium chitosan chloride nanoparticles	178	Ovalbumin	72.60	Mice	Elicited (specific); saliva and vaginal secretions	Elicited (specific); IgG1 dominantly	[106]
Mannosylated chitosan nanoparticles	400	pPES gene of *Mycobacterium tuberculosis*	−	Mice	Elicited (specific); bronchoalveolar lavage fluids	Elicited (specific)	[109]
